# The French eHealth Acceptability Scale Using the Unified Theory of Acceptance and Use of Technology 2 Model: Instrument Validation Study

**DOI:** 10.2196/16520

**Published:** 2020-04-15

**Authors:** Meggy Hayotte, Pierre Thérouanne, Laura Gray, Karine Corrion, Fabienne d'Arripe-Longueville

**Affiliations:** 1 Laboratoire Motricité Humaine Expertise Sport Santé Université Côte d'Azur Nice France; 2 Laboratoire d'Anthropologie et de Psychologie Cliniques, Cognitives et Sociales Université Côte d'Azur Nice France

**Keywords:** telemedicine, validation study, factor analysis, statistical, surveys and questionnaires, acceptability

## Abstract

**Background:**

Technology-based physical activity suggests new opportunities for public health initiatives. Yet only 45% of technology interventions are theoretically based, and the acceptability mechanisms have been insufficiently studied. Acceptability and acceptance theories have provided interesting insights, particularly the unified theory of acceptance and use of technology 2 (UTAUT2). In several studies, the psychometric qualities of acceptability scales have not been well demonstrated.

**Objective:**

The aim of this study was to adapt the UTAUT2 to the electronic health (eHealth) context and provide a preliminary validation of the eHealth acceptability scale in a French sample.

**Methods:**

In line with the reference validation methodologies, we carried out the following stages of validating the scale with a total of 576 volunteers: translation and adaptation, dimensionality tests, reliability tests, and construct validity tests. We used confirmatory factor analysis to validate a 22-item instrument with 7 subscales: Performance Expectancy, Effort Expectancy, Social Influence, Facilitating Conditions, Hedonic Motivation, Price Value, and Habit.

**Results:**

The dimensionality tests showed that the bifactor confirmatory model presented the best fit indexes: χ^2^_173_=434.86 (*P*<.001), χ^2^/*df*=2.51, comparative fit index=.97, Tucker-Lewis index=.95, and root mean square error of approximation=.053 (90% CI .047-.059). The invariance tests of the eHealth acceptability factor structure by sex demonstrated no significant differences between models, except for the strict model. The partial strict model demonstrated no difference from the strong model. Cronbach alphas ranged from .77 to .95 for the 7 factors. We measured the internal reliability with a 4-week interval. The intraclass correlation coefficients for each subscale ranged from .62 to .88, and there were no significant differences in the t tests from time 1 to time 2. Assessments for convergent validity demonstrated that the eHealth acceptability constructs were significantly and positively related to behavioral intention, usage, and constructs from the technology acceptance model and the theory of planned behavior.

**Conclusions:**

The 22-item French-language eHealth acceptability scale, divided into 7 subscales, showed good psychometric qualities. This scale is thus a valid and reliable tool to assess the acceptability of eHealth technology in French-speaking samples and offers promising avenues in research, clinical practice, and marketing.

## Introduction

### Background

Technology-based interventions to promote healthy behavior have been an emerging field of research for the past 10 to 20 years [1,2]. Among the healthy behaviors that are promoted, technology-based physical activity has brought to light new opportunities for public health interventions [3]. Several studies have evaluated the prospects of technologies such as exergames and active videogames [4], virtual reality [5], wearable physical activity trackers [6], website-delivered physical activity interventions [7], mobile phone apps [8,9], and video conferencing [10]. Electronic health (eHealth) physical activity promotion technologies have been designed not only for healthy adults [8], but also for vulnerable people in health care contexts, including cancer survivors [11,12], those in need of treatment for overweight and obesity [13,14] or cardiac rehabilitation [15], and older people [16,17]. All these technologies are popular (ie, positive assessment by many), with promising and reported positive outcomes [11-17]. However, the phenomena of usage cessation and losses to follow-up (ie, the law of attrition) are common problems [18]. Moreover, only 45% of the technology interventions are theoretically based, and the acceptability mechanisms have been insufficiently studied [1,19].

Acceptability and acceptance theories have provided interesting insights [20,21] into why some tools are chosen, accepted, and used more than others. The literature on acceptability and acceptance has emerged in different fields (eg, ergonomics, social psychology, management science) [22]. However, the acceptability and acceptance concepts have not been formally defined [23], and the distinction between the two has been based on the temporality of usage [21]. Acceptability refers to the a priori perceived use, whereas acceptance refers to the actual use [22]. Based on the proposed definitions [23], we define acceptability in this paper as the psychological antecedents of the behavioral intention to use technology without experience of the system. In the field of social psychology, the theory of reasoned action and the theory of planned behavior (TPB) [24,25] hold that attitudes and representations determine behavioral intention and real behavior [22]. These theories are the foundation for the technology acceptance model (TAM) [26], the most frequently used model in health informatics [27]. Nevertheless, several extensions have been proposed—TAM2 [28] and TAM3 [29]—revealing that the original TAM was not optimal in eHealth [27]. The unified theory of acceptance and use of technology (UTAUT), particularly its extension, UTAUT2, is today the most complete model, as it combines theory of reasoned action, TAM, a motivational model, TPB, a combined TPB and TAM, a model of personal computer use, diffusion of innovations theory, and social cognitive theory [29-31]. The UTAUT2 comprises 26 items divided into 8 constructs: Performance Expectancy (PE, 3 items), Effort Expectancy (EE, 4 items), Social Influence (SI, 3 items), Facilitating Conditions (FC, 4 items), Hedonic Motivation (HM, 3 items), Price Value (PV, 3 items), Habit (HT, 3 items), and Behavioral Intention (BI, 3 items).

Acceptability assessments in several studies in eHealth contexts have been based on tools without or with only partially demonstrated psychometric qualities [32,33]. However, to ensure the quality of future research, it is necessary to have scales with validated psychometric qualities [34]. The UTAUT model can be considered as a relevant framework for assessing the acceptability of eHealth, particularly for patient-centered assessment [20]. Yet, for theoretically based technologies in the health and wellness field, only 2 studies have been based on the UTAUT model [1], and 2 were conducted in France [27]. The scarcity French studies [1,27] may be due to the absence of validated scales in French to evaluate acceptability. To our knowledge, validated scales in the French language have been based on the TAM model [35] or on other definitions of acceptability in which the concept of acceptability is merged with the definition of usability [22,36]. The UTAUT2 [31] has already been translated into other languages (eg, German [37], Turkish [38], and Portuguese [39]) and has proven its validity; however, the psychometric qualities of the scales have been only partially demonstrated.

### Objective

The aims of this study were to adapt the UTAUT2 [31] to the eHealth context and to validate this version, which we called the eHealth acceptability scale, in French-speaking samples. This validated tool would allow for the development of further studies in this field.

## Methods

### Study Design

In line with the guidelines for scale validation from Vallerand et al [40] and Boateng et al [41], we conducted successive stages: translation and adaptation, dimensionality tests, reliability tests, and construct validity tests.

We managed the administration of the scale using LimeSurvey CE, version 2.06+ (LimeSurvey CE). We distributed the scale link by email or face-to-face at the end of students’ courses. We also distributed the link by email to health professionals and adults registered for adapted physical activity. In addition, we posted the link online via social media networks.

### Study Population

We recruited participants in various categories of the general population: students (studying sports, psychology, management, and computer science at a university in the South of France), health professionals (in the field of obesity), and adults with health conditions (ie, diabetes, cardiovascular disease, and obesity) registered for adapted physical activity sessions. To conduct the successive stages of validation, we divided the participants into 5 sample groups.

This study was approved by the French National Commission for Information Technology and Civil Liberties (authorization no: UCA-E18-00), and all participants gave their electronic consent before participation.

### Measures

#### Sociodemographics

The sociodemographic information, provided by all participants after they had completed the scale items, included their sex, year of birth, education level, and professional status.

#### eHealth Acceptability Scale

The UTAUT2 [31], originally developed in English in the field of mobile internet use, has 2 sections, 1 for the UTAUT2 scale comprising 26 items divided into 8 constructs, and the other for assessing the usage frequency of various apps for mobile internet. According to the definition we chose, acceptability corresponds to the psychological antecedents of the behavioral intention to use technology without experience of the system. Based on this definition, we excluded BI from the eHealth acceptability scale.

We produced this French adaptation of the UTAUT2 scale using the back-translation method [42]. Original items were translated individually by 4 researchers in the field of psychology and compiled to obtain a single French version. This French version was back-translated by 4 researchers unaware of the original version. The back-translators were subsequently asked to compare their own translation with the original to specify the differences. Differences were noted for 2 items, which were adjusted with the same procedure until all back-translators concluded that there was no difference. We then used a committee approach to replace mobile internet with a global expression that would include all the eHealth apps. We chose information and communication technologies for health, abbreviated as ICT for health, in reference to the wording used in a similar French questionnaire [36].

The preliminary version of the eHealth acceptability scale comprised 23 items divided into 7 subscales: PE (3 items), EE (4 items), SI (3 items), FC (4 items), HM (3 items), PV (3 items), and HT (3 items). Participants answered on a 7-point scale with labeled anchors ranging from 1, “strongly disagree,” to 7, “strongly agree*.*” We chose this 7-point scale because the participants were not familiar with the study context [43]. We administered this preliminary version of the eHealth acceptability scale to samples 1 to 3 and its adjusted form after the first confirmatory factor analysis (CFA) to samples 4 and 5. Sample 5 participants completed the scale a second time after 4 weeks for the test-retest reliability assessments.

#### Behavioral Intention

BI comes from the original UTAUT2 [31]. The 3 items were translated following the same procedure described above. The participants in all samples answered on a 7-point scale with labeled anchors ranging from 1, “strongly disagree,” to 7, “strongly agree.” BI was theoretically positively related to the constructs of the eHealth acceptability scale.

#### Usage

We measured usage in all samples as the frequency of eHealth technology use on a 7-point scale ranging from 1, “never,” to 7, “many times per day,” for 5 technologies: mobile health apps, forums or social networks for health, videos for health management, exergames or active video games, and health trackers. Usage was theoretically positively related to the constructs of the eHealth acceptability scale, especially FC and HT [31].

#### Technology Acceptance Model Constructs

Perceived Ease of Use (PEOU, 5 items), extracted from the TAM [26,35], was theoretically positively related to the constructs of the eHealth acceptability scale, especially EE [30]. This subscale was measured on a 7-point scale with labeled anchors ranging from 1, “strongly disagree,” to 7, “strongly agree.” Only the sample 4 participants completed this subscale to test for convergent validity.

#### Theory of Planned Behavior Constructs

Subjective Norms (SN, 3 items) and Perceived Behavioral Control (PBC, 5 items) extracted from the TPB [44,45] were theoretically positively related to the constructs of the eHealth acceptability scale, especially SI and FC [30]. These subscales were measured on 7-point scales with labeled anchors ranging from 1, “strongly disagree,” to 7, “strongly agree.” Only the sample 4 participants completed these subscales to test for convergent validity.

### Statistical Analyses

We performed all statistical analyses with IBM SPSS version 23 (IBM Corporation) and IBM SPSS Amos version 23 (IBM Corporation). We examined the missing data trends. The cutoff for an acceptable percentage of missing data has not been well established in the literature [46]. However, 5% is considered inconsequential [47], and the risk of statistical bias is considered when the rate is higher than 10% [48]. In our global sample, the missing rate was under 10%. For structural equation modeling, the maximum likelihood estimation and the multiple imputation for handling missing data presented close to equivalent good properties [49]. We applied the maximum likelihood estimations (considered the standard for structural equation models [46]) to be used in Amos v23.

#### Tests of Dimensionality

We ran tests of dimensionality using maximum likelihood estimation CFA in structural equation modeling according to several models [50]. We used the following indicators to assess competence of the model fit [51-54]: chi-square (significant values *P*≤.05), chi-square over degrees of freedom (significant values ≤3.00), comparative fit index (CFI; value >.90), Tucker-Lewis index (TLI; value >.90), root mean square error of approximation (RMSEA; value <.08), and the 90% confidence interval of RMSEA (ranging from .00 to .08).

We computed invariance of the eHealth acceptability scale between the sexes according to Gregorich’s methodology [55]. In the CFA framework, we tested a hierarchy of hypotheses to increasingly constrain the model. These hypotheses included configural (ie, no constraint), metric (ie, equal loads), strong (ie, equal covariances), and strict (ie, equal residuals) factorial invariance multigroup comparisons [55]. In addition to the previous indicators, we used the Akaike information criterion, expected cross-validation index, delta χ²/*df* (Δχ²/*df*), delta CFI (ΔCFI), and delta RMSEA (ΔRMSEA). Nonsignificant Δχ²/*df*, CFI differences <.01, and RMSEA differences <.015 indicated that the invariance hypothesis was not rejected [51,56].

#### Tests of Reliability

We calculated Cronbach alpha coefficients [57] to assess the internal consistency of each subscale; a value >.70 is considered satisfactory and a value >.60 is considered marginally acceptable [58]. We measured the test-retest reliability twice with an acceptable interval of 4 weeks [59] and a minimum sample size of 50 as recommended [60]. Data analyses involved the calculation of intraclass correlation coefficients (ICCs), the 95% confidence interval of the ICCs, and paired-sample *t* tests. We expected ICCs >.60 and the absence of significant differences in the *t* tests [40].

#### Tests of Construct Validity

We used Pearson correlation coefficients to measure the association between variables for the analysis of convergent validity. A significant correlation of .30 between the scale and each of the other theoretically appropriate measures was required [61].

## Results

### Study Population

To conduct the successive stages of validation, we divided the participants into 5 samples. Samples 1 (n=20), 2 (n=10), 3 (n=227), and 4 (n=319) were independent groups, and sample 5 (n=61) was a subgroup of sample 4. The global sample included 576 volunteers, mainly students (n=349, 60.6%), with 53.5% men (n=303) and a mean age of 26.8 (SD 10.9) years. We excluded 18 volunteers because they had never used eHealth technology. Table 1 presents detailed participant characteristics for each sample.

**Table 1:** Sociodemographic characteristics in each sample (N=576).

Characteristics			Sample 1 (n=20), n (%)		Sample 2 (n=10), n (%)		Sample 3 (n=227), n (%)		Sample 4 (n=319), n (%)		Sample 5^a^ (n=61), n (%)
**Age group, years**											
	18-24	3 (15.0)		2 (20.0)		128 (56.4)		238 (74.6)		25 (41.0)	
	25-34	8 (40.0)		5 (50.0)		46 (20.3)		41 (12.9)		13 (21.3)	
	≥35	9 (45.0)		3 (30.0)		43 (18.9)		38 (11.9)		23 (37.7)	
	Missing data	0		0		10 (4.4)		2 (0.6)		0	
**Sex**											
	Female	8 (40.0)		5 (50.0)		117 (51.5)		132 (41.4)		40 (65.6)	
	Male	12 (60.0)		5 (50.0)		100 (44.1)		186 (58.3)		21 (34.4)	
	Missing data	0		0		10 (4.4)		1 (0.3)		0	
**Education, years**											
	<12	7 (35.0)		2 (20.0)		1 (0.4)		0		0	
	12	5 (25.0)		4 (40.0)		125 (55.1)		143 (44.8)		9 (14.8)	
	15	6 (30.0)		1 (10.0)		30 (13.2)		116 (36.4)		19 (31.1)	
	≥17	2 (10.0)		3 (30.0)		61 (26.9)		60 (18.8)		33 (54.1)	
	Missing data	0		0		10 (4.4)		0		0	
**Professional status**											
	Unemployed	0		0		5 (2.2)		7 (2.2)		0	
	Student	3 (15.0)		8 (80.0)		103 (45.4)		235 (73.7)		25 (41.0)	
	Employed	15 (75.0)		0		103 (45.4)		74 (23.2)		36 (59.0)	
	Retired	2 (10.0)		2 (20.0)		6 (2.6)		3 (0.9)		0	
	Missing data	0		0		10 (4.4)		0		0	

^a^Sample 5 was a subsample of sample 4.

### Translation and Adaptation

We performed the first content clarity analysis on sample 1 (n=20), which revealed an acceptable clarity score (mean range from 4.40 to 7.00; mean 6.22, SD 0.71). Only 3 items (ie, EE2, SI2, and SI3) obtained a score of less than 5, which we rephrased according to participants’ suggestions. We performed a second content clarity analysis on sample 2 (n=10) regarding the 3 rephrased items. The clarity score increased for 2 items (SI2: mean range 4.40 to 6.20; SI3: mean range 4.90 to 6.20) but decreased for the third (EE2: mean range 4.65 to 3.30). We retained the 2 items with increased clarity scores in their rephrased form and the item with a decreased clarity score in its original translated wording. Multimedia Appendix 1 shows the preliminary pool of 23 items with their mean clarity scores.

### Tests of Dimensionality

We conducted a first maximum likelihood CFA on sample 3 (n=227) with the 23-item and 7-factor model. Standardized factor loadings were all higher than the recommended value of .50 [62], except for item FC4, for which the factor loading was .27. As a result, we removed item FC4. We conducted a second CFA using sample 4 (n=319) with the 22-item (ie, without FC4 item) and 7-factor correlated model (χ^2^_188_=471.80, *P*<.001). Fit indexes were as follows: χ²/*df*=2.51, CFI=.94, TLI=.91, and RMSEA=.069 (90% CI 0.061-0.077), revealing an acceptable model fit, with good standardized factor loadings for all items (ie, ≥0.63).

Based on the recommendations of Myers et al [50], we examined several models to assess the dimensionality of the scale, using samples 3 and 4 merged (n=546). Table 2 presents model fit indexes for each model. First, the unidimensional model did not present good fit indexes. Second, the first-order all-factor correlated model presented good fit indexes, as previously demonstrated. Third, the hierarchical second-order model presented acceptable fit indexes. Fourth, the bifactor confirmatory model presented the best fit indexes: χ²_173_=434.86 (*P*<.001), χ²/*df*=2.51, CFI=.97, TLI=.95, and RMSEA=.053 (90% CI .047-.059). These results sustained the possibility of extracting a global acceptability score from the scale.

**Table 2:** Fit indexes of the structural equation models (n=546).

Models	χ²	χ² *df*	*P* value	RMSEA^a^ (90% CI)	TLI^b^	CFI^c^	Δχ²	Δχ² *df*	Δ*P*
Unidimensional	4721.73	209	<.001	.199 (.194-.204)	.27	.39	N/A^d^	N/A	N/A
First-order all-factor correlated	532.29	188	<.001	.058 (.052-.064)	.94	.95	4189.44	21	<.001
Hierarchical second-order	825.98	202	<.001	.075 (.070-.081)	.90	.92	293.69	14	<.001
Bifactor confirmatory	434.86	173	<.001	.053 (.047-.059)	.95	.97	391.12	29	<.001

^a^RMSEA: root mean square error of approximation.

^b^TLI: Tucker-Lewis index.

^c^CFI: comparative fit index.

^d^N/A: not applicable.

We tested the invariance of the scale factorial structure following Gregorich’s recommendations [55], with samples 3 and 4 merged (n=535; 11 without sex information). The invariance tests were based on multigroup comparisons: female group (n=249) and male group (n=286). Each group presented good fit indexes for the CFA model (Table 3). We tested invariance in the 22-item 7-factor correlated model. Dimensional, metric, strong, and strict models presented good fit indexes (ie, CFI, TLI, and RMSEA) with significant chi-square *P* values (ie, *P*<.001). No significant differences between models were reported, except for the strict model (Table 3). A partial strict model, unconstrained for error of measurement for items EE2 and HM1, showed good fit indexes with no significant difference from the strong model.

**Table 3:** Fit indexes of structural modeling to assess sex invariance (n=535).

Models	χ²	χ² *df*	*P* value	RMSEA^a^	TLI^b^	CFI^c^	ECVI^d^	AIC^e^	Δχ²	Δχ² *df*	Δ*P*	ΔCFI	ΔRMSEA
Male (n=286)	427.42	188	<.001	.067	.93	.94	2.11	601.42	N/A^f^	N/A	N/A	N/A	N/A
Female (n=249)	330.71	188	<.001	.055	.96	.96	2.04	504.71	N/A	N/A	N/A	N/A	N/A
Dimensional^g^	758,12	376	<.001	.044	.94	.95	1.91	1018.12	N/A	N/A	N/A	N/A	N/A
Metric^h^	770.21	389	<.001	.043	.94	.95	1.88	1004.21	12.09	13	.520	0	0.001
Strong^i^	801.51	417	<.001	.042	.95	.95	1.84	979.51	31.30	28	.304	0	0.001
Strict^j^	915.77	439	<.001	.045	.94	.94	1.97	1049.77	114.26	22	<.001	0.012	0.002
Partial strict^k^	908.84	438	<.001	.044	.94	.94	1.91	1015.86	43.40	41	.370	0.011	0.002

^a^RMSEA: root mean square error of approximation.

^b^TLI: Tucker-Lewis index.

^c^CFI: comparative fit index.

^d^ECVI: expected cross-validation index.

^e^AIC: Akaike information criterion.

^f^N/A: not applicable.

^g^No invariance.

^h^Equal loads.

^i^Equal covariances.

^j^Equal residuals.

^k^Equal residuals except for items EE2 and HM1.

### Tests of Reliability

Cronbach alphas ranged from .77 to .95 in samples 3 and 4 (n=546) for the 7 eHealth acceptability factors (ie, α_PE_=.84; α_EE_=.88; α_SI_=.95; α_FC_=.78; α_HM_=.92; α_PV_=.86; α_HT_=.77) and were .93 for BI and .60 for usage.

We measured test-retest reliability in sample 5 (n*=*61) twice with an acceptable interval of 4 weeks [59]. Table 4 presents the results of the ICC and *t* tests. The ICCs for each construct ranged from .62 to .88. Thus, there were no significant differences in the *t* tests from time 1 to time 2.

**Table 4:** Descriptive statistics for the test-retest reliability in sample 5 (n=61).

Items	Score, mean (SD)		*t* test^a^	*P* value	ICC^b^ (95% CI)	*P* value
	Time 1	Time 2				
Performance Expectancy	4.67 (1.45)	4.46 (1.40)	t_60_=1.74	.09	.88 (.80-.93)	<.001
Effort Expectancy	5.43 (1.14)	5.60 (1.16)	t_60_=–1.22	.23	.74 (.57-.84)	<.001
Social Influence	3.67 (1.39)	3.52 (1.66)	t_60_=0.87	.39	.77 (.62-.86)	<.001
Facilitating Conditions	5.82 (0.92)	5.83 (1.09)	t_60_=–0.04	.97	.62 (.38-.78)	<.001
Hedonic Motivation	5.16 (1.29)	5.10 (1.21)	t_60_=0.46	.65	.80 (.67-.88)	<.001
Price Value	4.42 (1.14)	4.45 (1.11)	t_60_=–0.22	.83	.62 (.36-.77)	<.001
Habit	3.27 (1.35)	3.28 (1.40)	t_60_=–0.07	.94	.77 (.61-.86)	<.001

^a^Paired-sample *t* test.

^b^ICC: intraclass correlation coefficient.

### Tests of Construct Validity

We assessed convergent validity using Pearson correlation coefficients in sample 4 (n=319). BI was related to the eHealth acceptability subscales in the expected directions, even though the effect sizes were small for EE, FC, and PV. Usage was related to HT as expected, but not with FC. PEOU, SN, and PBC were related to the eHealth acceptability subscales in the expected directions. We observed additional significant correlation coefficients between constructs. Table 5 presents the complete matrix.

**Table 5:** Matrix of Pearson correlations in sample 4 (n=319)^a,b^.

Items	PE^c^	EE^d^	SI^e^	FC^f^	HM^g^	PV^h^	HT^i^	BI^j^	Usage	PEOU^k^	PBC^l^	SN^m^
PE	N/A^n^	N/A	N/A	N/A	N/A	N/A	N/A	N/A	N/A	N/A	N/A	N/A
EE	*.33*	N/A	N/A	N/A	N/A	N/A	N/A	N/A	N/A	N/A	N/A	N/A
SI	*.54*	.14	N/A	N/A	N/A	N/A	N/A	N/A	N/A	N/A	N/A	N/A
FC	.12	*.62*	.12	N/A	N/A	N/A	N/A	N/A	N/A	N/A	N/A	N/A
HM	*.40*	*.47*	.21	*.40*	N/A	N/A	N/A	N/A	N/A	N/A	N/A	N/A
PV	.24	.23	.19	.28	.28	N/A	N/A	N/A	N/A	N/A	N/A	N/A
HT	*.57*	.21	*.54*	N/A	.30	.22	N/A	N/A	N/A	N/A	N/A	N/A
BI	*.58*	.27	*.52*	.22	*.45*	.22	*.65*	N/A	N/A	N/A	N/A	N/A
Usage	*.43*	.24	.30	.12	.28	N/A	*.50*	*.46*	N/A	N/A	N/A	N/A
PEOU	.21	*.64*	.15	*.59*	*.36*	*.31*	.19	.26	.17	N/A	N/A	N/A
PBC	*.31*	*.43*	.22	*.49*	.30	.30	*.31*	*.41*	*.32*	*.51*	N/A	N/A
SN	*.47*	.13	*.71*	.13	.24	.25	*.52*	*.55*	*.36*	.24	*.31*	N/A

^a^Significant correlations between subscales (ie, >.30) [61] are shown in italics.

^b^Shows only correlations with *P*<.05.

^c^PE: Performance Expectancy.

^d^EE: Effort Expectancy.

^e^SI: Social Influence.

^f^FC: Facilitating Conditions.

^g^HM: Hedonic Motivation.

^h^PV: Price Value.

^i^HT: Habit.

^j^BI: Behavioral Intention.

^k^PEOU: Perceived Ease of Use.

^l^PBC: Perceived Behavioral Control.

^m^SN: Subjective Norms.

^n^N/A: not applicable.

## Discussion

### Principal Findings

This study aimed to fill a gap in the acceptability literature by providing a validated scale based on the UTAUT2 model [31] that would be suitable for eHealth contexts in French-speaking samples. The eHealth acceptability scale comprised 22 items divided into 7 subscales: PE (3 items), EE (4 items), SI (3 items), FC (3 items), HM (3 items), PV (3 items), and HT (3 items).

The dimensionality tests showed that the first-order all-factor correlated model and the bifactor confirmatory model had good fit indexes. The results confirmed the possibility of both using the subscales individually and extracting a global score of acceptability. The internal consistency evaluated by Cronbach alphas was considered satisfactory [57] and thus was confirmed. The ICCs for each subscale were above .60 and there were no significant differences in the *t* test over a 4-week period. These results demonstrated the temporal stability of the eHealth acceptability scale. Although it might seem important to attain strict factorial invariance, practical experience suggests that this is almost unachievable [55]. The partial strict factorial invariance pointed to the sex invariance in our analysis. This conclusion was one of the major findings, as it confirms that the eHealth acceptability scale can be used in male and female French-speaking samples.

Convergent validity assessments showed that subscales of the eHealth acceptability scale were significantly positively related to BI, usage, and the PEOU construct from the TAM [26], and significantly positively related to the SN and PCB constructs from the TPB [44]. These preliminary results need to be confirmed in future studies.

The strength of this scale validation was that it followed all the steps recommended by Boateng [41].

### Limitations

Some limitations must nevertheless be acknowledged. One of these limitations, as in all rating scales, is the self-reported nature of the responses, which can be biased based on social desirability [63]. Another limitation is the homogeneity of the samples we used. Most of the participants were young and students. Few participants with low socioeconomic status were included, which limited generalizability. In populations that are not familiar with eHealth tools, it may be necessary to deliver specific education, notably by providing a description of the terms used. In addition, given the age distribution of our sample (ie, centered on ages 18-34 years), we could not test the age invariance. Furthermore, the simultaneous modification of the language (ie, into French) and the context (ie, adaptation to eHealth) may have led to potential interactions and is a limitation. The study would probably have been stronger if we had validated a French-language instrument before changing the context.

### Comparison With Prior Work

Compared with the English-language UTAUT2 model [31], the French eHealth acceptability scale comprised 22 items divided into 7 subscales: PE (3 items), EE (4 items), SI (3 items), FC (3 items), HM (3 items), PV (3 items), and HT (3 items), according to our analyses. We removed item FC4 for its inconsistency; the low loading was also observed to a lesser extent in the German translation [37], although not removed. The sex invariance demonstrated in our analysis was not provided in the original version [31], nor in the other translations [37,38].

### Future Directions

In future studies, it will be necessary to test the constructs of the eHealth acceptability scale, which was based on the UTAUT2 model, in French samples and to estimate the explained variance in BI and usage. In addition, evaluation of age invariance will be necessary. The suggested adaptation to the eHealth context could also be replicated in other languages. Specifically, an English validation of the eHealth acceptability scale would be of interest in order to provide a common tool across French- and English-speaking samples. This scale could be used in future research to identify acceptability correlates in different contexts. It could also be used in clinical practice before implementing a new technology in health care or in the field of marketing as new technologies are developed.

### Conclusions

We designed a 22-item French-language eHealth acceptability scale, divided into 7 subscales. The scale demonstrated good psychometric qualities (ie, reliability, dimensionality, validity). With this preliminary validation, the scale can be used with men and women to assess the acceptability of eHealth technology in French-speaking samples and offers promising avenues in research, clinical practice, and marketing.

## Appendix

Multimedia Appendix 1 Preliminary version of the eHealth acceptability scale and adapted items of the UTAUT2.

## Abbreviations

BI: Behavioral Intention
CFA: confirmatory factor analysis
CFI: comparative fit index
EE: Effort Expectancy
eHealth: electronic health
FC: Facilitating Conditions
HM: Hedonic Motivation
HT: Habit
ICC: intraclass correlation coefficient
PBC: Perceived Behavioral Control
PE: Performance Expectancy
PEOU: Perceived Ease of Use
PV: Price Value
RMSEA: root mean square error of approximation
SI: Social Influence
SN: Subjective Norms
TAM: technology acceptance model
TLI: Tucker-Lewis index
TPB: theory of planned behavior
UTAUT: unified theory of acceptance and use of technology
