# Supplementary material for: The French eHealth Acceptability Scale Using the Unified Theory of Acceptance and Use of Technology 2 Model: Instrument Validation Study
Source: J Med Internet Res. 2020 Apr 15;22(4):e16520. doi: 10.2196/16520 (PMC7191343; doi:10.2196/16520)
Supplement: Multimedia Appendix 1 [file jmir_v22i4e16520_app1.docx]

Appendix 1. Preliminary version of the eHealth acceptability scale and adapted items of the UTAUT2 ^a-b^

| **Dimensions** | **Codes** | **Translated and adapted items of the UTAUT2-eHealth** | **Clarity scores (M)** |
| --- | --- | --- | --- |
| Performance Expectancy  (PE) | |  |  |
|  | PE1. | Je trouve les TIC pour la santé utiles dans ma vie quotidienne.  *I find ICT for Health useful in my daily life.* | 6.4 |
|  | PE2. | Utiliser les TIC pour la santé m’aide à accomplir les choses plus rapidement.  *Using ICT for Health helps me accomplish things more quickly.* | 6.6 |
|  | PE3. | Utiliser les TIC pour la santé augmente ma productivité.  *Using ICT for Health increases my productivity.* | 6.2 |
| Effort Expectancy  (EE) | |  |  |
|  | EE1. | Apprendre comment utiliser les TIC pour la santé est facile pour moi.  *Learning how to use ICT for Health is easy for me.* | 6.7 |
|  | EE2. | Mon interaction avec les TIC pour la santé est claire et compréhensible.  *My interaction with ICT for Health is clear and understandable.* | 4.7 |
|  | EE3. | Je trouve les TIC pour la santé faciles à utiliser.  *I find ICT for Health easy to use.* | 6.9 |
|  | EE4. | Il est facile pour moi de devenir habile dans l’utilisation des TIC pour la santé.  *It is easy for me to become skillful at using ICT for Health.* | 5.7 |
| Social Influence  (SI) | |  |  |
|  | SI1. | Les personnes qui sont importantes pour moi pensent que je devrais utiliser les TIC pour la santé.  *People who are important to me think that I should use ICT for Health.* | 5.9 |
|  | SI2. | Les personnes de mon entourage pensent que je devrais utiliser les TIC pour la santé.  *People who influence my behavior think that I should use ICT for Health.* | 6.2 |
|  | SI3. | Les personnes dont l’avis compte pour moi souhaitent que j’utilise les TIC pour la santé.  *People whose opinions that I value prefer that I use ICT for Health.* | 6.2 |
|  | *(Continued on next page)* | | |

Appendix 1. Preliminary version of the eHealth acceptability scale and adapted items of the UTAUT2^a-b^ (continued)

| **Dimensions** | **Codes** | | **Translated and adapted items of the UTAUT2-eHealth** | **Clarity scores (M^c^)** |
| --- | --- | --- | --- | --- |
| Facilitating Conditions  (FC) | | |  |  |
|  | | FC1. | J’ai les ressources nécessaires pour utiliser les TIC pour la santé.  *I have the resources necessary to use ICT for Health.* | 6.0 |
|  |  | FC2. | J’ai les connaissances nécessaires pour utiliser les TIC pour la santé.  *I have the knowledge necessary to use ICT for Health.* | 6.8 |
|  |  | FC3. | Les TIC pour la santé sont compatibles avec les autres technologies que j’utilise.  *ICT for Health is compatible with other technologies I use.* | 6.8 |
|  |  | FC4.^d^ | Je peux obtenir de l’aide des autres lorsque j’ai des difficultés à utiliser les TIC pour la santé.  *I can get help from others when I have difficulties using ICT for Health.* | 6.5 |
| Hedonic Motivation  (HM) | | |  |  |
|  | HM1. | | Utiliser les TIC pour la santé est amusant.  *Using ICT for Health is fun.* | 7.0 |
|  | HM2. | | Utiliser les TIC pour la santé est agréable.  *Using ICT for Health is enjoyable.* | 6.9 |
|  | HM3. | | Utiliser les TIC pour la santé est divertissant.  *Using ICT for Health is very entertaining.* | 6.8 |
| Price Value  (PV) | | |  |  |
|  | PV1. | | Les TIC pour la santé sont à un prix raisonnable.  *ICTs for Health are reasonably priced.* | 6.9 |
|  | PV2. | | Les TIC pour la santé ont un bon rapport qualité-prix.  *ICTs for Health are good value for the money.* | 6.9 |
|  | PV3. | | Au prix actuel, les TIC pour la santé valent le coût.  *At the current price, ICTs for Health provide good value.* | 5.6 |
| Habit  (HT) | | |  |  |
|  | HT1. | | L’utilisation des TIC pour la santé est devenue une habitude pour moi.  *The use of ICT for Health has become a habit for me.* | 6.8 |
|  | HT2. | | Je suis « accro » à l’utilisation des TIC pour la santé.  *I am addicted to using ICT for Health.* | 6.1 |
|  | HT3. | | Je dois utiliser les TIC pour la santé.  *I must use ICT for Health.* | 5.9 |

^a^Adapted items of the UTAUT2 are in italics.

^b^For each item, the participant had to answer on a 7-point scale ranging from (1) *strongly disagree* to (7) *strongly agree.*

^c^M: mean clarity scores.

^d^Deleted items following the first CFA.
